# Preparation and Electrochemical Characterization of Si@C Nanoparticles as an Anode Material for Lithium-Ion Batteries via Solvent-Assisted Wet Coating Process

**DOI:** 10.3390/nano12101649

**Published:** 2022-05-12

**Authors:** Jongha Hwang, Mincheol Jung, Jin-Ju Park, Eun-Kyung Kim, Gunoh Lee, Kyung Jin Lee, Jae-Hak Choi, Woo-Jin Song

**Affiliations:** 1 Department of Polymer Science and Engineering, Chungnam National University, Daejeon 34134, Korea; jongha3238@o.cnu.ac.kr (J.H.); mnb7809@o.cnu.ac.kr (M.J.); wlswnsla09@gmail.com (J.-J.P.); dmsrud11kr14@gmail.com (E.-K.K.); 2 Department of Chemical Engineering and Applied Chemistry, Chungnam National University, Daejeon 34134, Korea; golee124@gmail.com (G.L.); kjlee@cnu.ac.kr (K.J.L.)

**Keywords:** silicon anode, carbon coating, lithium-ion batteries, phenolic resin

## Abstract

Silicon-based electrodes are widely recognized as promising anodes for high-energy-density lithium-ion batteries (LIBs). Silicon is a representative anode material for next-generation LIBs due to its advantages of being an abundant resource and having a high theoretical capacity and a low electrochemical reduction potential. However, its huge volume change during the charge–discharge process and low electrical conductivity can be critical problems in its utilization as a practical anode material. In this study, we solved the problem of the large volume expansion of silicon anodes by using the carbon coating method with a low-cost phenolic resin that can be used to obtain high-performance LIBs. The surrounding carbon layers on the silicon surface were well made from a phenolic resin via a solvent-assisted wet coating process followed by carbonization. Consequently, the electrochemical performance of the carbon-coated silicon anode achieved a high specific capacity (3092 mA h g^−1^) and excellent capacity retention (~100% capacity retention after 50 cycles and even 64% capacity retention after 100 cycles at 0.05 C). This work provides a simple but effective strategy for the improvement of silicon-based anodes for high-performance LIBs.

## 1. Introduction

The development of high-tech electric vehicles (EVs) and energy storage systems (ESSs) requires a light and high-performance energy storage device [1,2]. Lithium-ion batteries (LIBs) are the representative energy devices for these large-scale applications due to their high energy density, high working voltage, and long-term stability [3,4,5]. However, in the case of EVs, commercialized EVs are not yet sufficient to replace gasoline-based vehicles owing to the impractical driving mileage that depends on the energy density of LIBs [4,6]. In accordance with the increasing energy needs, commercial combinations of active materials such as a carbon anode (graphite, 372 mA h g^−1^) and a lithium metal oxide cathode (LCO, 274 mA h g^−1^), which are currently commercially available, need to be replaced with high-capacity materials [7]. Therefore, the development of high-capacity active materials has been a major focus of recent battery research trends [8].

Silicon (Si), which is naturally abundant and has a high theoretical capacity (3579 mA h g^−1^ at room temperature), has drawn significant attention as a promising alternative anode material for high-energy-density LIBs [9,10,11]. Despite its distinguishing features mentioned above, Si undergoes a huge volume change (>300%) during the charge–discharge cycles, causing unstable solid electrolyte interface (SEI) formation and structural pulverization of the electrode, finally leading to poor cycling performance. To effectively address the large volume change of Si, a variety of strategies such as morphology control, polymeric binder design, and surface coating methods have been presented [12,13]. Among them, the surface coating approach is considered as one of the most favorable methods, including the molten salt, combustion synthesis, and template assembly strategies, because it can be mass produced as well as guaranteeing a stable electrochemical performance as it stabilizes the SEI layer in cycles [14,15,16,17,18,19].

Herein, we report a simple but effective strategy for carbon coating on the surface of silicon nanoparticles (Si NPs) using a low-cost phenolic resin to obtain high-performance anodes for LIBs. A phenolic resin as a carbon precursor has several advantages, such as the high carbonization yield and low cost. We used the solvent-assisted wet process method to achieve the homogeneous coating of phenolic resin-based organic layers on the surface of the Si NPs. After the carbonization process of the phenolic resin-coated Si NPs (Si@resin NPs), carbon layers were successfully formed on the surface of the Si NPs. We optimized the ratio of the carbon content and Si NPs to achieve both a high cycle stability and high capacity of the Si-based anode. In particular, the carbon-coated Si NPs (Si@C NPs) were not detached from the electrode during the repeated lithiation/delithiation process over 100 cycles, even though the Si NPs without carbon layers easily collapsed after 50 cycles. These results reveal that the surface coating approach using a phenolic resin has the potential to develop high-performance silicon-based anodes for LIBs.

## 2. Materials and Methods

### 2.1. Synthesis of Si@C NPs

Commercial Si NPs which have a diameter of around 50 nm were purchased from Nanostructured & Amorphous Materials, Inc., Houston, TX, USA. A phenolic resin (KPH-F2001, HiRENOL) was obtained from Kolon Industries, Inc., Seoul, Korea. Acetone as a solvent was purchased from Samjeon Pure Chemical Co., Seoul, Korea. Si@C NPs were synthesized using the following procedure. An amount of 3 g of commercial Si NPs and different amounts (0.30, 0.75, 3.0 g) of the phenolic resin were dissolved in 400 mL acetone under continuous ultrasonic stirring at room temperature (25 °C) for 3 h. After stirring, the acetone was evaporated in a thermostat oven at a temperature of 40 °C for 12 h. The obtained Si@resin NPs were transferred to a tube furnace and carbonized under a nitrogen atmosphere at 1000 °C for 1 h with a heating rate of 5 °C min^−1^ to obtain core–shell Si@C NPs. The carbon content of the Si@C NPs was controlled by using different amounts of the phenolic resin. The obtained Si@C NPs were labeled Si_10_@C_1_, Si_4_@C_1_, and Si_1_@C_1_ according to the ratio of Si to the phenolic resin.

### 2.2. Characterization

The morphology of the samples was investigated by transmission electron microscopy (TEM) and scanning electron microscopy (SEM). To prepare the samples for the TEM measurement, the powdered samples were fully dispersed in ethanol in an ultrasonic bath and then dried on a copper grid. Raman spectra were recorded on a Raman spectrometer (Bruker Korea Office Co., Seongnam, Korea) with a He–Ne laser wavelength of 632.8 nm. To measure the carbon content of the Si@C NPs, thermogravimetric analysis (TGA, N-1000, Scinco Co., Seoul, Korea) was carried out from 0 to 900 °C in air with a heating rate of 10 °C min^−1^. Fourier-transform infrared spectroscopy (FTIR) was performed to examine the chemical interaction of the samples (Spectrum Two, Perkin Elmer Korea Office Co., Daejeon, Korea).

### 2.3. Electrochemical Measurements

The working electrode was prepared by mixing 60 wt% of Si@C NPs (active material), 20 wt% of Super P (conducting agent, Wellcos, Gunpo, Korea), and 20 wt% of poly (acrylic acid) (binder, Acros Organics, Geel, Belgium) in deionized water. Then, the mixed slurry was coated onto a copper foil using a doctor blade, which was 40 µm thick. The coated electrode was dried in an oven at 70 °C for 30 min, and in a vacuum oven at 80 °C for 2 h, and then cut into disks with a 14 mm diameter. The mass loading of all active materials was about 0.6 mg cm^−2^. Coin-type cells (CR2032) were assembled in a glove box under an argon atmosphere. Lithium foil with a thickness of 300 μm was employed as the counter and reference electrodes, which were parted by a polypropylene separator (Cellgard 2400, Wellcos, Gunpo, Korea) in a coin cell. For the preparation of the electrolyte, 1 M LiPF_6_ was dissolved in dimethyl carbonate (EC), and diethyl carbonate (DEC) which had a volume ratio of 3:7 with the addition of 10 wt% fluoroethylene carbonate (FEC). The first galvanostatic charge–discharge voltage profiles of the half-cell were measured at a rate of 0.05 C, and the cycle performances of the cells were obtained at a rate of 0.5 C (2.3 A g^−1^ of the current density at a rate of 1 C) for 100 cycles between 0.05 V and 1.0 V in a constant-temperature chamber at 25 °C using a battery measurement system (WBCS 3000, WonATech, Seoul, Korea). Electrochemical impedance spectroscopy (EIS) was also tested at a 10 mV amplitude signal in the frequency range from 500 kHz to 0.1 Hz.

## 3. Results and Discussion

**Figure 1:**
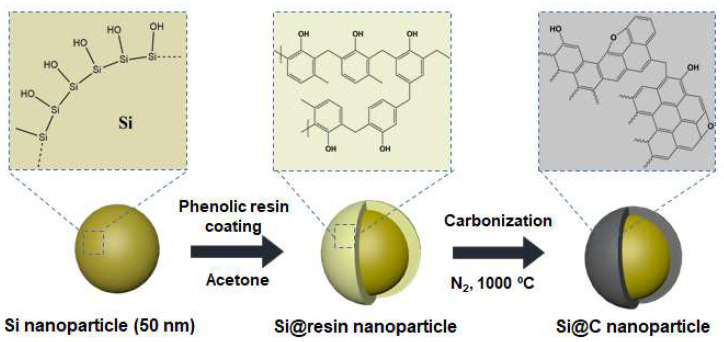
Schematic process of the phenolic resin-derived carbon coating method on Si NPs.

For the uniform carbon layer on the 50 nm Si NPs, we utilized a phenolic resin as the carbon coating precursor via the solvent-assisted wet process. Figure 1 shows the schematic fabrication process of the phenolic resin-derived Si@C NPs as anode materials for LIBs. In the wet process for coating the phenolic resin on the surface of the Si NPs, the silanol groups in the native oxide layer of the Si NPs strongly interacted with the hydroxy groups of the phenolic resin via hydrogen bonding, as shown in the second step of Figure 1. Before the carbonization process, the phenolic resin was uniformly coated on the silicon surface after the drying process of the solvent in Figure S1 in the Supporting Information. The obtained Si@resin NPs were carbonized under a nitrogen atmosphere at 1000 °C for 1 h to convert the resin coating layer into a carbon coating layer. The carbon shells surrounding the silicon cores can block the direct contact of the electrolytes with the Si NPs for the prevention of the huge volume expansion during the lithiation/delithiation process and also enhance the electrical conductivity of the Si NPs for the fast charge transport [20].

**Figure 2:**
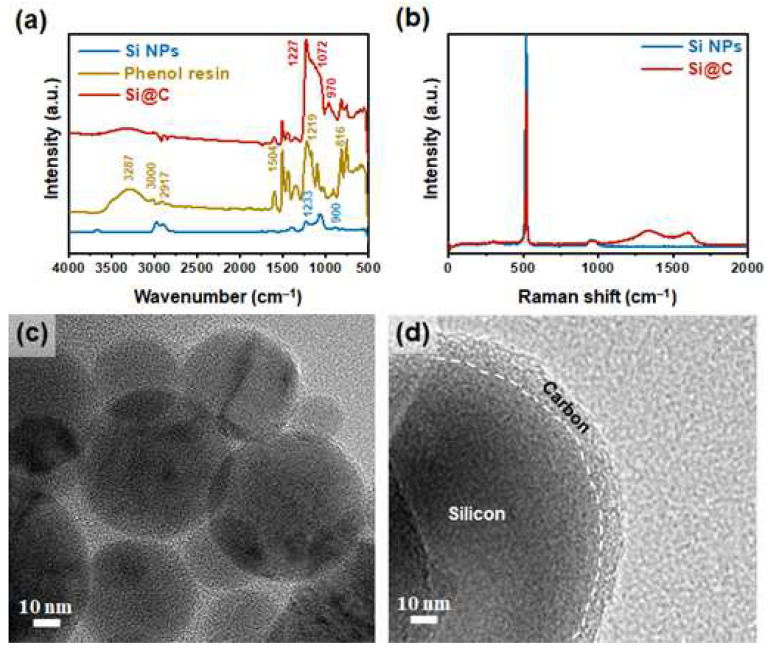
(**a**) FT−IR spectra of Si NPs, phenolic resin, and Si@C; (**b**) Raman spectra of Si and Si@C NPs; (**c**,**d**) TEM images of Si@C.

Changes in the chemical structures after coating the phenolic resin on the Si NPs were observed by FT-IR spectroscopy, and the results are shown in Figure 2a. The FT-IR spectrum of the Si NPs showed characteristics peaks at 1233 and 900 cm^−1^, corresponding to the Si-O-Si and Si-OH groups, respectively [21]. After coating the phenolic resin on the surface of the Si NPs, characteristic peaks for the phenolic resin also appeared at 3000–3620 cm^−1^ (aromatic OH groups), 2840 cm^−1^ (aliphatic C-H groups), 1504 cm^−1^ (aromatic ring), and 1227 cm^−1^ (phenol-O groups). Therefore, these results confirm that the phenolic resin was successfully coated on the surface of the Si NPs through this process [22].

The conversion of the coated phenol resin into a graphitic carbon layer by the carbonization process was investigated by Raman analysis. The Si@C NPs showed two additional characteristic peaks for the D and G bands at 1351 cm^−1^ and 1580 cm^−1^, corresponding to the amorphous/disordered and crystalline/graphitic carbon structures, respectively, as shown in Figure 2b. The value (I_D_/I_G_) of the intensity ratio of the D band to the G band was 1.1, which indicates structural defects in the carbon structure. In addition, the intensity of the characteristic band for the Si NPs at around 518 cm^−^^1^ decreased, revealing the well-sealed core–shell structure of the Si@C NPs [23].

To confirm the structure of the Si@C NPs, we checked the TEM images as shown in Figure 2c,d. The results reveal that the Si@C NPs had typical core–shell structures and that the uniform and continuous carbon layers were coated on the surface of the Si NPs. These NPs were of sphere-like shapes with an average diameter of around 40 nm; meanwhile, the thickness of the carbon shell was around 10 nm. Figure 2d and Figure S2 shows that the silicon cores were successfully encapsulated by the amorphous carbon shell [24]. The crystalline structures of the Si and Si@C NPs were further investigated by XRD analysis (Figure S3). The Si@C showed the same distinct peaks corresponding to the (111), (220), (311), (400), (331), and (422) planes for the Si NPs. However, the Si@C showed the weak peak for at 22.8°, attributed to the (002) plane of the amorphous carbon layers (Figure S3) [16]. The results of the Raman, TEM, and XRD analyses revealed that the amorphous carbon layers were formed on the surface of the Si cores via wet coating followed by carbonization.

**Figure 3:**
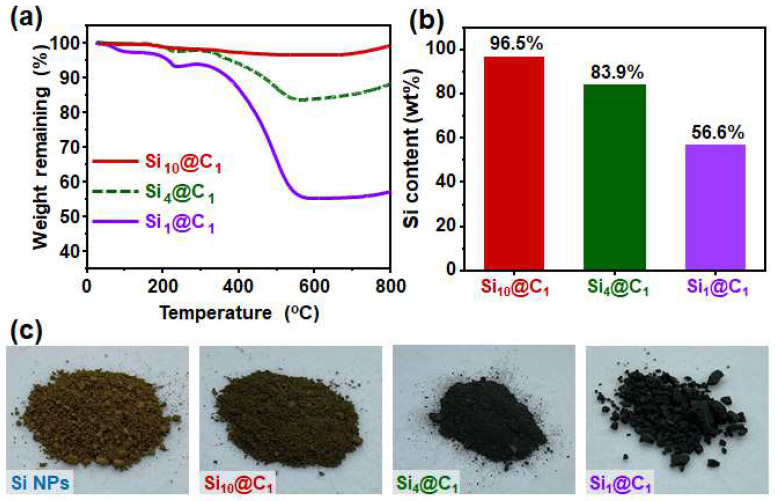
(**a**) TGA curves of Si@C NPs derived from various ratios of Si NPs and phenolic resin; (**b**) Si contents of Si@C NPs; (**c**) photo images of Si and Si@C NPs.

The Si contents of the Si@C NPs were determined by TGA analysis. As observed in Figure 3a, there was a small peak for some weight gain around 250 °C before the start of the degradation. This trend was due to the oxidation reactions of the coated phenolic resin [25]. Thereafter, the weight loss originated from the carbon combustion in the range of 300 to 550 °C. The weight of the silicon-containing samples slightly increased because of the oxidation of silicon at the high temperature, as shown in Figure 3a [16]. Figure 3b reveals that the silicon contents of the Si, Si_10_@C_1_, Si_4_@C_1_, and Si_1_@C_1_ NPs were 100%, 96.5%, 83.9%, and 56.6%, respectively [26]. The pristine Si NPs were a brown powder consisting of spherical NPs with a dominant size of 40 nm. After coating with different carbon contents, the color of the products was changed from brown to dark green and finally to black, depending on the carbon contents, as shown in Figure 3c.

**Figure 4:**
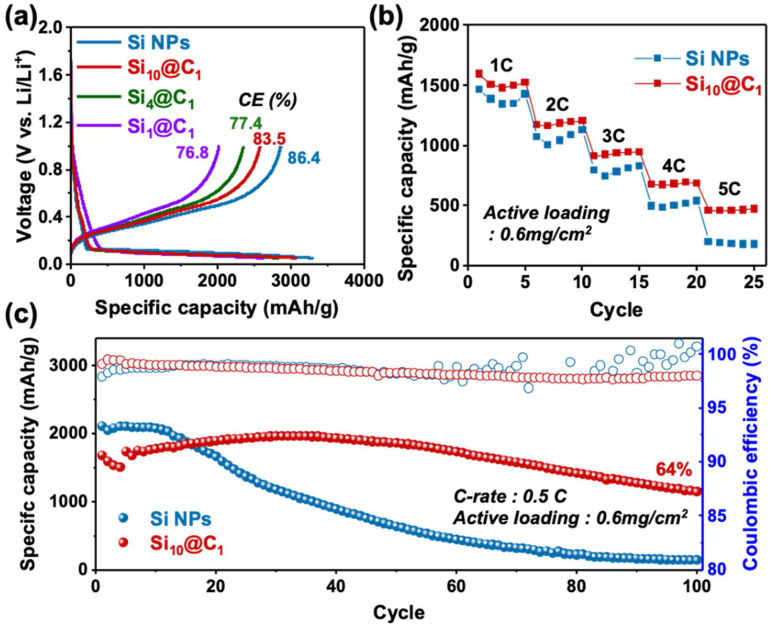
(**a**) Galvanostatic charge–discharge profiles for the Si@C∥Li half-cell; (**b**) the specific capacities of the Si- and Si_10_@C_1_-based half-cells at various C rates ranging from 1 C to 5 C; (**c**) cycling performance of the Si- and Si_10_@C_1_-based electrodes at a rate of 0.5 C.

**Figure 5:**
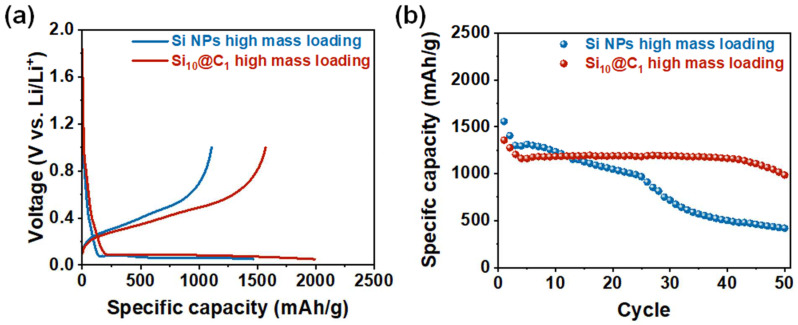
Electrochemical performances of the Si@C‖Li half-cell with a high mass loading (~1.2 mg cm^−2^): (**a**) galvanostatic charge–discharge profiles of the SiNPs and Si@C electrode with a high mass loading; (**b**) cycle performance of the SiNPs and Si@C electrode with a high mass loading at a rate of 0.5 C.

To confirm the electrochemical property of the carbon contents derived from the various ratios of the phenolic resin and 50 nm Si NPs, the first galvanostatic charge–discharge curves of the Si@C-based electrodes with the Si, Si_10_@C_1_, Si_4_@C_1_, and Si_1_@C_1_ NPs were tested at a rate of 0.05 C with a cut-off voltage of 0.05 and 1 V at room temperature (25 °C) in a coin-type lithium half-cell with a mass loading of 0.6 mg cm^−2^. All the calculations of the capacity in the lithium half-cell were based on the total weight of the active materials. The discharge capacity of the Si, Si_10_@C_1_, Si_4_@C_1_, and Si_1_@C_1_-based electrodes was 3318, 3092, 2840, and 2637 mA h g^−1^ at 0.05 C, respectively. The capacity of Si_10_@C_1_ was much higher than that of Si_4_@C_1_ and Si_1_@C_1_, which implies that the high contents of Si led to the high capacity. The coulombic efficiency of the Si, Si_10_@C_1_, Si_4_@C_1_, and Si_1_@C_1_-based half-cells was 86.4%, 83.5%, 77.4%, and 76.8%, respectively. As the carbon contents of the Si@C NPs increased, the coulombic efficiency gradually decreased because the carbon layer on the Si NPs irreversibly reacted with the lithium at a low potential [20], which is confirmed in Figure 4a. Si_1_@C_1_ showed a side reaction from 0.1 to 0.8 V, which was greater than that of Si, Si_10_@C_1_, and Si_4_@C_1_. In addition, the interfacial resistance of Si_4_@C_1_ and Si_1_@C_1_ was 12.5 Ω and 22 Ω, respectively, which was higher than that of Si10@C1 (10.04 Ω), as shown in Figure S4. Therefore, we decided on the Si1@C10 electrode as an optimized electrode with a high capacity and coulombic efficiency. To further investigate the electrochemical property of the optimized Si_10_@C_1_ and Si NP electrodes, the rate performance was investigated ranging from 1 C to 5 C, as shown in Figure 4b. The discharge capacity and capacity retention of the half-cell with the Si NP electrode were 1466, 1072, 798, 497, and 203 mA h g^−1^ at 1 C, 2 C, 3 C, 4 C, and 5 C, and 73.1, 54.4, 33.9, and 13.8% at 2 C, 3 C, 4 C, and 5 C, respectively. In contrast, the discharge capacity and capacity retention of the half-cell with the Si_10_@C_1_ electrode were 1596, 1172, 914, 676, and 459 mA h g^−1^ at 1 C, 2 C, 3 C, 4 C, and 5 C, and 73.4, 57.2, 42.4, and 28.8% at 2 C, 3 C, 4 C, and 5 C, respectively. Compared to the capacity retention of the half-cell with the Si NPs at 5 C, the rate capability of the half-cell with the Si_10_@C_1_ electrode was more than twice that of the Si NPs (13.8% vs. 28.8%). These results indicate that the carbon coating method on the surface of the Si NPs using a phenolic resin achieved a high rate capability because the carbon layer alleviated the charge transfer between the Si NPs and electrolytes. As shown in Figure 4c, we also examined the cycling performance of the Si_1_@C_1_ electrode at a rate of 0.05 C for 100 cycles. The coulombic efficiency of the Si NP electrodes was unstable after 50 cycles, indicating that it was difficult for the Si NPs to maintain their original structure during the lithiation/delithiation process [25]. However, the Si_10_@C_1_ electrode accomplished almost 100% capacity retention after 50 cycles and even 64% capacity retention after 100 cycles with 98% coulombic efficiency. Significantly, the silicon particles were not detached from the Si@C electrode after 100 cycles, although the Si NPs without carbon layers easily collapsed after 50 cycles (Figures S5 and S6 in the Supporting Information). The cycle stability and coulombic efficiency can be improved through the optimization of the carbon contents of the Si@C NPs. We also tested the electrode with our stable silicon anode at a higher mass loading (~1.2 mg cm^−2^), as shown in Figure 5. The discharge capacity of our carbon-coated silicon electrode with the high mass loading accomplished 1989 mA h g^−1^, and the initial coulombic efficiency was 78.9% after the first cycle (Figure 5a). These values were higher than those of the silicon-based electrode (1467 mA h g^−1^ and 75.5%) without the carbon coating. We also confirmed the cycle performance for 50 cycles. The capacity retention of the Si@C electrode with the high mass loading was 72.6% after 50 cycles, which was much higher than the 26.9% capacity retention of the silicon electrodes (Figure 5b).

**Figure 6:**
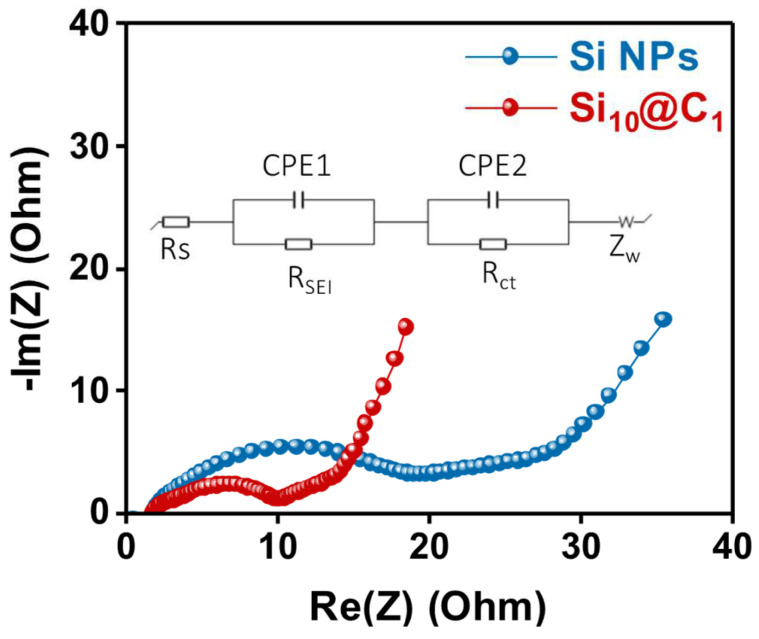
Nyquist plot of the Si- and Si_10_@C_1_-based electrodes.

To understand the excellent performance of the Si@C NPs as an active material, we performed an EIS analysis. Figure 6 shows the Nyquist plots containing the information on the electrolyte resistance at a high-frequency region (R_s_), the interfacial resistance on an anode electrode (R_SEI_), the charge transfer resistance at a low-frequency region (R_ct_), and the Warburg diffusional impedance [27]. The first semi-circle is related to the interfacial resistance (R_SEI_), and the second semi-circle presents the charge transfer resistance (R_ct_). As shown in Figure 6, the R_s_ of the Si_10_@C_1_ electrode was 1.84 Ω, similar to that of the Si NPs (1.82 Ω). However, the R_SEI_ and R_ct_ of Si_10_@C_1_ were much lower than those of the Si NPs (R_SEI_: 10.04 vs. 19.46 Ω, R_ct_: 3.51 vs. 7.68 Ω, respectively), which implies that the carbon layer on the silicon surface reduced the interfacial resistance between the Si@C NPs and electrolytes (Table S1 in the Supporting Information). The CV curve was tested in the range from 0 V to 1.0 V at a 0.1 mV scan rate (Figure S7 in the Supporting Information). The cathodic peak was revealed at 0.19 V in the CV curves, revealing that the lithiation process properly occurred during the formation of the Li-Si alloy between the crystalline Si and Li^+^ ions. The two anodic peaks of the delithiation process were also confirmed at 0.37 V and 0.52 V by the phase transition from the Li-Si alloy to amorphous silicon [28]. The electrochemical performance of the Si@C NPs prepared in this study was comparable to or even superior to previously reported various carbon-coated silicon anodes (Table S2).

## 4. Conclusions

In conclusion, we constructed core–shell Si@C NPs for high-performance LIBs using a phenolic resin as the carbon coating precursor through the solvent-assisted wet process. Our strategy for the carbon coating method using phenolic resin realizes an improvement in the interfacial resistance and a stable lithiation/delithiation process of silicon-based anodes. These excellent electrochemical properties result in outstanding cycle stability (~100% capacity retention after 50 cycles and even 64% capacity retention after 100 cycles at 0.05 C) and high coulombic efficiency (98% coulombic efficiency after 100 cycles at 0.05 C). The carbon coating method with phenolic resin represents an effective method to obtain high-performance silicon-based anodes for LIBs.

## Data Availability

The data presented in this study are available on request from the corresponding author.

## Supplementary Materials

The following Supporting Information can be downloaded at: https://www.mdpi.com/article/10.3390/nano12101649/s1, Figure S1: The SEM image of Si NPs and phenolic resin-coated Si NPs; Figure S2: High resolution TEM images of (a,b) Si@C; Figure S3: XRD data of Si NPs and Si@C; Figure S4: EIS data of Si_10_@C_1_, Si_4_@C_1_, and Si_1_@C_1_; Figure S5: The SEM image of Si NPs electrode and Si_10_@C_1_ electrode after 100 cycles; Figure S6: The photo image of Si and Si_10_@C_1_ electrodes after 100 cycles; Figure S7: The cyclic voltammetry curves of Si@C anode; Table S1: EIS data of Si NPs and Si_10_@C_1_; Table S2: Comparison of electrochemical performance of various carbon-coated silicon anodes. Refs. [29,30,31,32] are cited in the Supplementary Material.
