# Supplementary material for: Preparation and Electrochemical Characterization of Si@C Nanoparticles as an Anode Material for Lithium-Ion Batteries via Solvent-Assisted Wet Coating Process
Source: Nanomaterials (Basel). 2022 May 12;12(10):1649. doi: 10.3390/nano12101649 (PMC9145801; doi:10.3390/nano12101649)
Supplement: Supplementary file 1 [file nanomaterials-12-01649-s001.zip › nanomaterials-1688297-supplementary.pdf]

nanomaterials

Supplementary Materials

Preparation and Electrochemical Characterization of Si@C Nanoparticles as an Anode Material for Lithium-Ion Batteries via Solvent-Assisted Wet Coating Process

Jongha Hwang [1,†], Mincheol Jung [1,†], Jin-Ju Park [1,†], Eun-Kyung Kim [1], Gunoh Lee [2], Kyung Jin Lee [2], Jae-Hak Choi [1,*] and Woo-Jin Song [1,*]

[1] Department of Polymer Science and Engineering, Chungnam National University, Daejeon 34134, Korea; jongha3238@o.cnu.ac.kr (J.H.); mnb7809@o.cnu.ac.kr (M.J.); wlswnsla09@gmail.com (J.-J.P.); ek_dmsrud11kr14@gmail.com (E.-K.K.)
[2] Department of Chemical Engineering and Applied Chemistry, Chungnam National University, Daejeon 34134, Korea; golee124@gmail.com (G.L.); kjlee@cnu.ac.kr (K.J.L.)
* Correspondence: jaehakchoi@cnu.ac.kr (J.-H.C.); wjsong@cnu.ac.kr (W.-J.S.)
† These authors have contributed equally to this work.

Citation: Hwang, J.; Jung, M.; Park, J.-J.; Kim, E-K.; Lee, G.; Lee, K.J.; Choi, J.-H.; Song, W.-J. Preparation and Electrochemical Characterization of Si@C Nanoparticles as an Anode Material for Lithium-Ion Batteries via Solvent-Assisted Wet Coating Process. *Nanomaterials* **2022**, *12*, 1627. https://doi.org/10.3390/nano12101627

Academic Editors: Zhipeng Sun, Bin Dong and Dong Xie

Received: 2 April 2022
Accepted: 5 May 2022
Published: 12 May 2022

Publisher's Note: MDPI stays neutral with regard to jurisdictional claims in published maps and institutional affiliations.

Copyright: © 2022 by the authors. Licensee MDPI, Basel, Switzerland. This article is an open access article distributed under the terms and conditions of the Creative Commons Attribution (CC BY) license (https://creativecommons.org/licenses/by/4.0/).

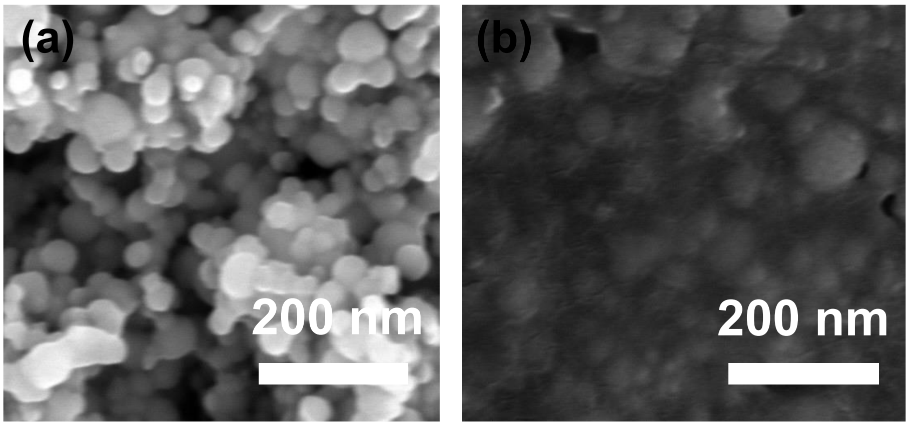

Figure S1. The SEM image of (**a**) Si NPs and (**b**) phenolic resin-coated Si NPs.

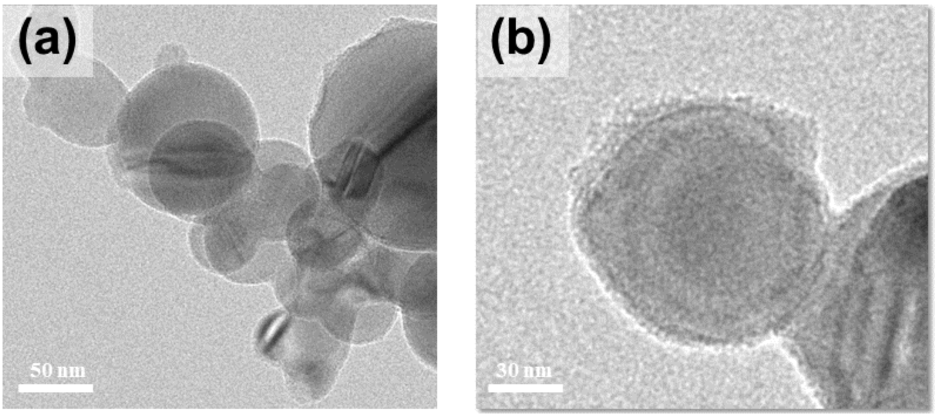

Figure S2. High resolution TEM images of (**a**,**b**) Si@C.

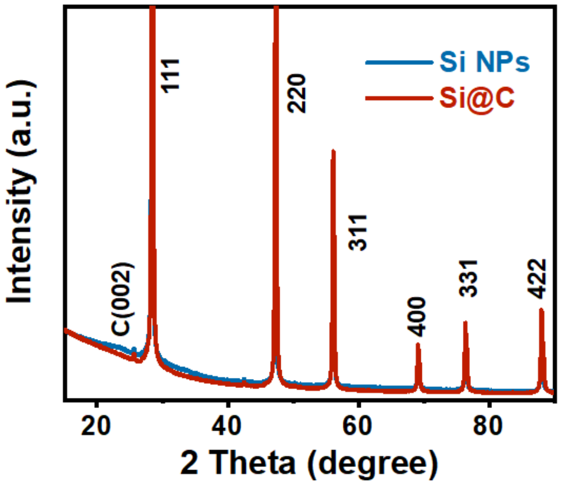

Figure S3. XRD data of Si NPs and Si@C.

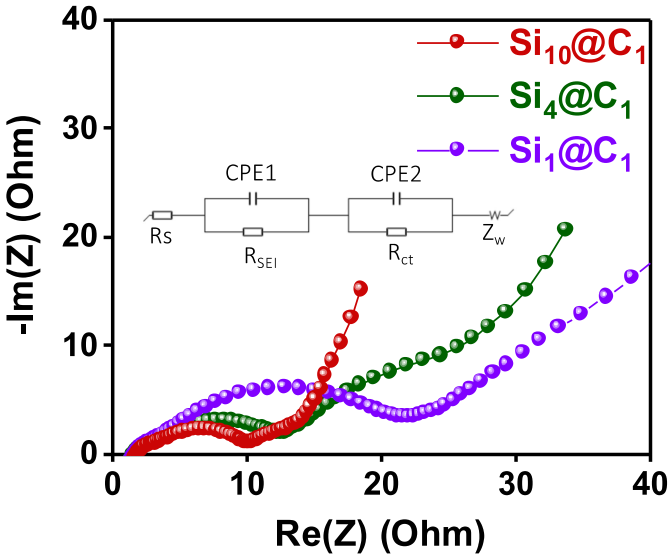

Figure S4. EIS data of $Si_{10}@C_1$, $Si_4@C_1$, and $Si_1@C_1$.

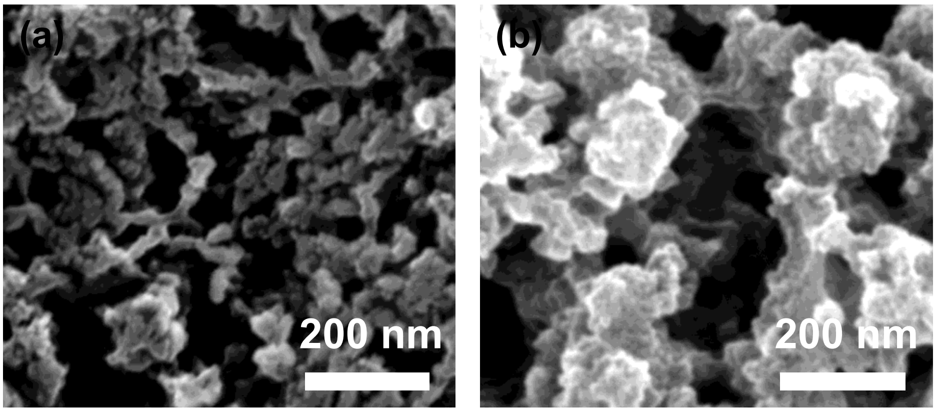

Figure S5. The SEM image of (**a**) Si NPs electrode and (**b**) $Si_{10}@C_1$ electrode after 100 cycles.

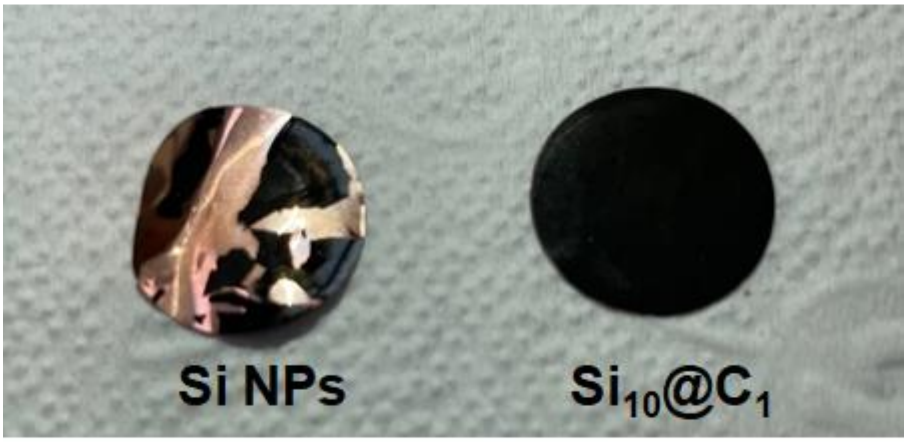

Figure S6. The photo image of Si and $Si_{10}@C_1$ electrodes after 100 cycles.

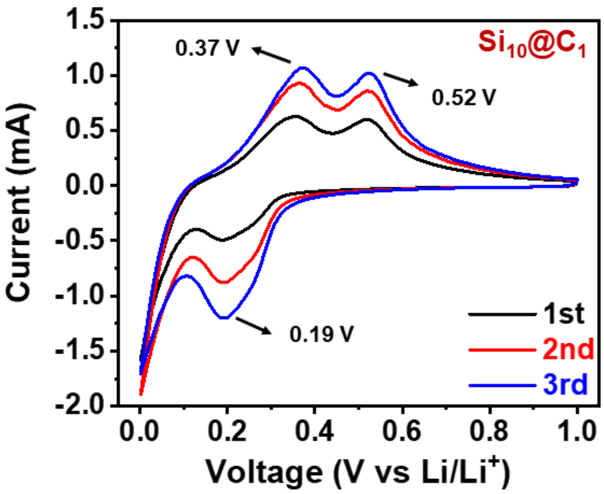

Figure S7. The cyclic voltammetry curves of Si@C anode.

Table S1. EIS data of Si NPs and $Si_{10}@C_1$.

Electrodes.	R_s	R_{SEI}	R_{ct}
**Si NPs**	1.82	19.46	7.68
**$Si_{10}@C_1$ NPs**	1.84	10.04	3.51

Table S2. Comparison of electrochemical performance of various carbon-coated silicon anodes.

Active Material	Carbon Precursors	Discharge Capacity (mA h g^{-1})	ICE (%)	Retention (%)/Cycles	Capacity after Cycles (mA h g^{-1})	Ref
**Si NPs (~50 nm)**	Phenolic resin	2283	66.7	86.3/50	1107	[1]
**Si NPs (~100 nm)**	Dopamine	2258	72.2	83/50	804	[2]
**Si NPs**	Graphene/ egg white	2169	86.2	56/200	1045	[3]
**Si NPs (50~100 nm)**	rGO	2200	61	63/200	968	[4]
**Si NPs (~50 nm)**	Reusable phenolic resin	3092	83.5	100/50	1140	Our work

Reference

1. Lu, Z.; Li, B.; Yang, D.; Lv, H.; Xue, M.; Zhang, C., A self-assembled silicon/phenolic resin-based carbon core–shell nanocomposite as an anode material for lithium-ion batteries. *RSC Adv.* **2018**, *8*, 3477–3482.
2. Pan, L.; Wang, H.; Gao, D.; Chen, S.; Tan, L.; Li, L.; Facile synthesis of yolk–shell structured Si–C nanocomposites as anodes for lithium-ion batteries. *Chem. Commun.* **2014**, *50*, 5878–5880.
3. Ou, J.; Jin, F.; Wang, H.; Wu S.; Zhang, H.; Carbon coated Si nanoparticles anchored to graphene sheets with excellent cycle performance and rate capability for Lithium-ion battery anodes, *Surf. Coat. Technol.* **2021**, *418*, 127262.
4. Wang, Q.; Meng, T.; Li, Y.; Yang. J.; Huang, B.; Ou, S.; Meng, C.; Zhang, S.; Tong, Y. Consecutive chemical bonds reconstructing surface structure of silicon anode for high-performance lithium-ion battery. *Energy Storage Mater*. **2021**, *39*, 354–364.
